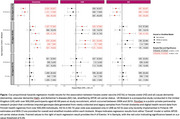# Associations between herpes zoster vaccination and herpes zoster with dementia risk in APOE‐e4 carriers and non‐carriers

**DOI:** 10.1002/alz70860_104034

**Published:** 2025-12-23

**Authors:** Jonathan Davitte, Usha Gungabissoon, Jessica Chao, Spiros Denaxas, Ian J Douglas, Ana Torralbo, Cai Ytsma, Chris Tomlinson, Natalie K Fitzpatrick, Jorge Esparza Gordillo, Andrew M Goldfine, Paul J Harrison, Maxime Taquet, Robert A Scott, John A Todd, Chun‐Fang Xu

**Affiliations:** ^1^ GSK, Collegeville, PA, USA; ^2^ GSK, London, United Kingdom; ^3^ Institute of Health Informatics, University College London, London, United Kingdom; ^4^ London School of Hygiene and Tropical Medicine, London, United Kingdom; ^5^ GSK, Tres Cantos, Madrid, Spain; ^6^ University of Oxford, Oxford Health NHS Foundation Trust, Oxford, United Kingdom; ^7^ GSK, Stevenage, United Kingdom; ^8^ Oxford Health Biomedical Research Centres, University of Oxford, Oxford, United Kingdom

## Abstract

**Background:**

Recent studies have reported a reduced risk of dementia in herpes zoster vaccine (HZ‐Vx) recipients. However, none evaluated whether genetic factors influence this potentially protective effect. We used the UK Biobank (UKB) and FinnGen (FG) cohorts to investigate associations between HZ‐Vx or herpes zoster (HZ; shingles) and dementia risk by APOE‐e4 carrier status.

**Method:**

This study used structured data from 1990‐2022 (UKB)/2023 (FG). Exposed adults (HZ‐Vx/HZ) were matched (1:5) by age, sex, and APOE‐e4 status (≥1 allele vs. 0) to unexposed adults. Observation started 12‐months post‐exposure. We measured associations between each exposure and incident all‐cause dementia (dementia), vascular dementia (VaD) or Alzheimer's disease (AD) by Cox regressions in age‐exposure‐specific cohorts: 1) HZ‐Vx in 65‐74‐year‐olds; 2) HZ in 45‐64‐year‐olds; and 3) HZ in 65‐74‐year‐olds. Hazard ratios (HRs) are reported overall and by APOE‐e4 status.

**Result:**

HZ‐Vx was associated with a reduced risk of dementia (HR=0.68 [95% confidence interval: 0.59‐0.77]) and AD (HR=0.69 [0.57‐0.84]) in UKB. Associations in APOE‐e4 non‐carriers and carriers were: dementia HR=0.59 [0.48‐0.73] and HR=0.75 [0.63‐0.90]; AD: HR=0.64 [0.47‐0.88] and HR=0.73 [0.57‐0.92], respectively. HZ in 45‐64‐year‐olds was associated with increased risk of dementia (UKB: HR=1.21 [1.04‐1.42]; FG: HR=1.55 [1.31‐1.82]) and VaD (UKB: HR=1.46 [1.07‐1.97]; FG: HR=1.78 [1.28‐2.46]). HZ in 45‐64‐year‐olds was associated with dementia (HR=1.43 [1.14,1.79]) and AD (HR=1.59 [1.10‐2.29]) in APOE‐e4 non‐carriers only in UKB (Figure); in FG, the associations were observed in both non‐carriers (HR=1.61 [1.31‐1.97]) and carriers (HR=1.45 [1.11‐1.90]) for dementia. HZ in 65‐74‐year‐olds was not associated with any outcome.

**Conclusion:**

Our findings support previous research indicating a reduced risk of dementia among HZ‐Vx recipients. We uniquely showed the potential effect in both APOE‐e4 carriers and non‐carriers. Conversely, we observed an increased risk of dementia in individuals with a recorded HZ diagnosis in the younger age group. Further research is required to understand the role of HZ as a potential risk factor for dementia.